# Characterization of A Three-Dimensional Organotypic Co-Culture Skin Model for Epidermal Differentiation of Rat Adipose-Derived Stem Cells

**DOI:** 10.22074/cellj.2016.4553

**Published:** 2016-08-24

**Authors:** Zeinab Ghanavati, Mahmoud Orazizadeh, Vahid Bayati, Mohammad Reza Abbaspour, Layasadat Khorsandi, Esrafil Mansouri, Niloofar Neisi

**Affiliations:** 1 Cellular and Molecular Research Center, Ahvaz Jundishapur University of Medical Sciences, Ahvaz, Iran; 2 Department of Anatomical Sciences, School of Medicine, Ahvaz Jundishapur University of Medical Sciences, Ahvaz, Iran; 3 Targeted Drug Delivery Research Center, School of Pharmacy, Mashhad University of Medical Sciences, Mashhad, Iran; 4 Department of Medical Virology, School of Medicine, Ahvaz Jundishapur University of Medical Sciences, Ahvaz, Iran

**Keywords:** Co-Culture Techniques, Fibroblasts, Mesenchymal Stem Cells, Polycaprolactone

## Abstract

**Objective:**

The organotypic co-culture is a well-known technique to examine cellular interactions and their roles in stem cell proliferation and differentiation. This study aims to evaluate the effects of dermal fibroblasts (DFs) on epidermal differentiation of adipose-derived stem cells (ASCs) using a three-dimensional (3D) organotypic co- culture technique.

**Materials and Methods:**

In this experimental research study, rat DFs and ASCs were isolated and cultured separately on electrospun polycaprolactone (PCL) matrices. The PCL matrices seeded by ASCs were superimposed on to the matrices seeded by DFs in order to create a 3D organotypic co-culture. In the control groups, PCL matrices seeded by ASCs were placed on matrices devoid of DFs. After 10 days, we assessed the expressions of keratinocyte-related genes by real-time reverse transcriptase-polymerase chain reaction (RT-PCR) and expression of pan-cytokeratin protein by immunofluorescence in the differentiated keratinocyte-like cells from co- culture and control groups. Keratinocyte-like cell morphologies were also observed by scanning electron microscopy (SEM).

**Results:**

The early, intermediate, and terminal differentiation keratinocyte markers-*Cytokeratin14, Filaggrin,* and *Involucrin* significantly expressed in the co-culture groups com- pared to the control ones (P<0.05). We observed pan-cytokeratin in keratinocyte-like cells of both groups by immunofluorescence. SEM observation of the co-culture groups showed that the differentiated keratinocyte-like cells developed a polygonal cobblestone shape, considered characteristic of keratinocytes.

**Conclusion:**

The 3D organotypic co-culture bilayered construct that consisted of DFs and ASCs was an effective technique for epidermal differentiation of ASCs. This co-culture might be useful for epidermal differentiation of stem cells for future applications in skin regeneration.

## Introduction

The skin, the largest tissue of the body, plays an essential role in protection against water loss, pathogens, and microorganisms (1,2). Skin loss may occur by thermal injury, trauma, and chronic ulcerations secondary to diabetes mellitus, venous stasis, and pressure (3). The increasing number of patients, risk of amputations, unsatisfactory results from existing therapies, and heavy socioeconomic burden cause chronic wounds to be a worldwide health and economic problem. Accordingly, development of tissue-engineered skin equivalents represents an innovative solution for treatment of non-healing skin wounds (4). The prospects for implanting normal cells from adult or fetal tissue or implanting genetically reconstituted cells from the same patient have generated a whole new branch of culture technique, that of tissue engineering (5,8). 

Tissue engineering encompasses the production of tissue equivalents by an organotypic culture technique (9,10), isolation and differentiation of human embryonic stem (ES) and adult totipotent stem cells [mesenchymal stem cells (MSCs)], gene transfer, materials science, utilization of bioreactors, and transplantation technology (11,13). The development of histotypic and organotypic culture techniques also increases the accuracy of *in vivo* modeling. Histotypic or histoculture is defined as the "tissue-like," culture of one cell type, whereas an organotypic culture technique implies the presence of more than one cell type that interact as they may in the organ of origin (i.e., skin) (14). The key event that distinguishes an organotypic culture from a histotypic culture is the introduction of heterotypic cell interaction, which includes diffusible paracrine effects and signaling that implicates the extracellular matrix (ECM). The relationship of the cells allows the generation of a structured microenvironment, cell polarity, and enhanced differentiation. The organotypic co-culture technique, as a powerful tool provides new prospects for the study of cell interaction among discrete, defined populations of homogeneous and potentially genetically and phenotypically defined cells (9,10). Tissue engineering is an interdisciplinary scientific approach that aims to reconstruct damaged tissues or organs to restore their functions (5). Tissue engineering has three crucial elements: cells, engineering materials [three-dimensional (3D) scaffold], and conducive physicochemical factors that allow for cell growth in 3D materials (6,8). It is important to emphasize that the skin is an appropriate model organ for testing novel concepts of tissue engineering (9). However, owing to the structural and functional complexity of the skin, engineering skin substitutes for normal skin remain challenging (10,11). Therefore, research in this area take priority over other areas of skin research. 

MSCs have received special attention in regenerative medicine research because they are multipotential and have high proliferative activity (13,15,16). Adipose-derived stem cells (ASCs) are an abundant source of stem cells, easily accessible and cultivated *in vitro*. These cells are prepared for tissue engineering approaches from different adipose tissue sources within the body (16,18). According to research, ASCs have interesting properties that make them useful as treatment for non-healing wounds such as enhancement of fibroblast and keratinocyte proliferation, remodeling of the ECM, induction of angiogenesis (19,21), and differentiation into keratinocytes (21,24). 

3D scaffolds have the capability to support *in vitro* tissue formation and maturation. Cell seeding onto a porous scaffold is a common strategy in tissue engineering (12,13). The 3D structure of biomaterial matrices provides a micro-environment for cell behaviors and allows intercellular interactions with more realistic, biochemical, and physiological manners (12). A 3D-nanofiber scaffold has been shown to act similar to the ECM/basement membrane and enhance the proliferation and selfrenewal of stem cells (6,8,11,13). 

This study aimed to investigate the effects of dermal fibroblasts (DFs) on epidermal differentiation of ASCs in a 3D organotypic co-culture skin model. In order to mimic the natural constitution of skin tissue, we cultured ASCs and DFs on separate electrospun polycaprolactone (PCL) matrices as epidermal and dermal equivalents, respectively. Subsequently, we developed a 3D organotypic co-culture skin model by placing the epidermal equivalent on top of the dermal one in order to assess the effect of DFs on ASCs differentiation toward keratinocyte-like cells. 

## Materials and Methods

### Fibroblast isolation and culture

We conducted this experimental research on 10 male Wistar rats (8 weeks old, weight: 150-200 g). Rats were obtained from the Laboratory Animal Research Center at Ahvaz Jundishapur University of Medical Sciences (AJUMS) and maintained under standard conditions with controlled temperature (23˚C) and a light/dark cycle (12/12 hour) in the Animal House at the Department of Anatomical Sciences, AJUMS. All experiments were performed in accordance with the protocols approved by the Institutional Animal Care and Use Committee and with the Guidelines for Care and Use of Experimental Animals required by AJUMS. DFs were isolated from the back skin of the rats as previously described with some modification (25,26). After cervical dislocation, the skin was shaved and twice sterilized by 70% ethanol for 2 minutes, followed by Betadine solution for 5 minutes. After excision of the skin tissue, the sample was immersed three times, for 5 minutes each, in sterile Ca^+2^/ Mg^+2^-free phosphatebuffered saline (PBS, pH=7.2, Gibco, USA), then minced into small pieces (5×5 mm^2^). The small samples were incubated overnight in 0.2% trypsin (Gibco, USA) at 4˚C. Then, the epidermis was carefully removed from the dermis with fine forceps. The dermis was minced lightly and digested with 0.1% collagenase type I (Sigma, USA) for 45-60 minutes at 37˚C. Afterwards, the suspension was strained through a 70 µm nylon cell strainer. Cells were centrifuged at 150×g for 5 minutes and re-suspended in proliferation medium that consisted of Dulbecco’s Modified Eagle Medium (DMEM, Gibco, USA) with 20% fetal bovine serum (FBS, Gibco, USA), 1% penicillin/streptomycin (P/S), and 2 mM L-glutamine (Gibco, USA), and incubated at 37˚C and 5% CO_2_. On the following days, the medium was replaced by fresh medium. Once confluent, the cells were passaged using 0.25% trypsin (Sigma, USA) that contained a 0.1% EDTA (Sigma, USA) solution. The heterogeneous dermal cell population or DFs were used from passages 3 to 5 (26). 

### Isolation and culture of adipose-derived stem cells

Stem cell isolation was carried out according to previous protocols with some modifications (27). Adipose tissue was isolated from the gonadal fat pads of Wistar rats, washed three times with PBS, minced into small pieces, and digested with 0.1% collagenase type I for 40 minutes. In order to separate stromal vascular fraction (SVF) cells from mature adipocytes, the cell suspension was centrifuged at 200×g for 5 minutes. The pellet was re-suspended in proliferation medium (DMEM-high glucose supplemented with 10% FBS, 1% P/S, and 2 mM Lglutamine). SVF cells were plated onto a 25 cm^2^cell culture flask at a density of 2.5×10^4^cells/ cm^2^and incubated at 37˚C in 5% CO_2_. After 48 hours, we removed the non-adherent cells and added fresh medium. After 4 passages, the cells were characterized by flow cytometry evaluations of their surface markers [CD44, CD73, and CD90 (as positive markers) and CD45 (as a negative marker)] and by testing their ability to differentiate into osteogenic and adipogenic lineages in individual differentiation medium, as previously reported (27). We determined that the cells were ASCs. These ASCs were used for the organotypic co-culture experiments. 

### Matrix preparation

PCL (Aldrich, Mw: 80000 g/mol) matrices were prepared by the electrospinning method (28). PCL was dissolved in 50:50 (v/v) mixtures of N-dimethylformamide and dichloromethane to obtain a 15% (w/v) PCL solution. The solution was electrospun in a 10 mL syringe located at a distance of 160 mm from the collector. The application of a 25 kv voltage between the needle tip and collector resulted in ejection of a fluid jet from the tip of the needle at a flow rate of 1 ml/hour. The solvent evaporated and PCL nanofibers were collected on an aluminum foil on the collector. The construct was dried overnight at room temperature. 

### Matrix characterization by scanning electron microscopy

After coating the PCL matrices with a thin gold-palladium alloy layer under vacuum, we used a scanning electron microscope (SEM, Zeiss Evo 50, Germany) to visualize morphology and surface topography of the electrospun fibers. Fiber diameters and size distribution were measured from the SEM images using Image J software version 1.46 (NIH, MD) (28). 

### Mechanical tensile test

We used a material testing machine (Wance, China) equipped with a 5 kN load cell to evaluate the mechanical properties of the electrospun PCL matrix. The samples were cut into 10 mm width×50 mm length (gauge length: 25 mm) strips. The crosshead speed in the tensile mode was set at 10 mm/minutes and the analyses were performed under ambient conditions. At least three samples were analyzed. Tensile strength, elastic modulus, and tensile strain were obtained from the stress-strain curves generated by the testing machine (28). 

### Organotypic co-culture

Before cell seeding, the matrices specimens were soaked in 70% ethanol for 1 hour for sterilization and rinsed with PBS. Next, specimens were incubated overnight in DMEM with 25% FBS to increase cell attachment. A total of 1×10^5^ASCs/cm^2^in fresh medium were seeded onto the PCL matrix. The ASCs/PCL constructs as epidermal equivalents were incubated overnight at 37˚C and 5% CO_2_to allow for cell adherence. In a similar manner, we seeded 2×10^4^DFs/cm^2^onto the PCL matrix with the same dimensions as the dermal equivalents. After 24 hours, epidermal equivalents were rinsed with PBS to remove unattached cells and carefully placed onto the dermal equivalents to create a 3D organotypic co-culture. We considered these to be the co-culture groups. 

**Fig.1:**
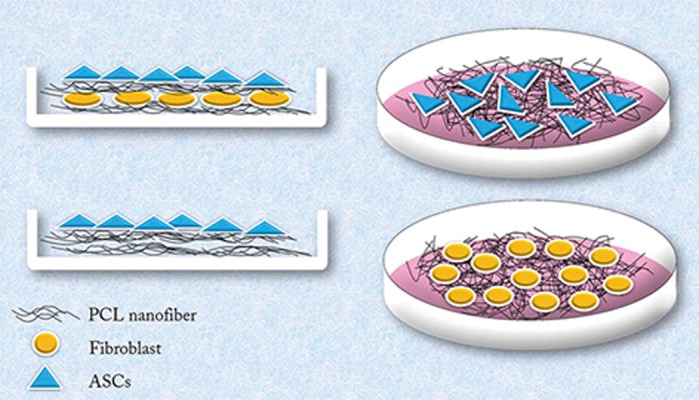
Schematic representation of adipose-derived stem cells (ASCs) and dermal fibroblasts (DFs) cultured on polycaprolactone (PCL) nanofibers and superimposition of nanofibers seeded with ASCs on DFs cultured on nanofibers. A. Co-culture (experimental) group and B. Control group.

The control groups consisted of epidermal equivalents placed on the PCL matrices without DFs (Fig.1). Thereafter, epidermal differentiation medium was added to these skin constructs (29) which consisted of DMEM supplemented with 10% FBS, 30 ng/ml epidermal growth factor (EGF, PeproTech Ltd., USA) and 10 ng/ml keratinocyte growth factor (KGF, PeproTech Ltd., USA) at 37˚C in an air-liquid interface for 10 days. The medium was changed every other day. We assessed epidermal gene expressions, cytokeratin protein synthesis and morphological evaluations in keratinocyte-like cells differentiated from ASCs on epidermal equivalents in both the co-culture and control groups. 

### Real-time reverse transcriptase-polymerase chain reaction analysis

Real-time RT-PCR was performed to confirm the expressions of *Cytokeratin14, Filaggrin,* and *Involucrin* with ABI (STEP1USA) according to the manufacturer’s instructions. The cells were lysed for RNA extraction using the RNeasy plus Mini Kit (Qiagen, Gaithersburg, MD, USA) by homogenizing cell-scaffold constructs in Eppendorf tubes. Cells were quantified by a NanoDrop 2000c spectrophotometer (Thermo Scientific, USA). cDNA synthesis was performed using a QuantiTect Reverse Transcription Kit (Qiagen, Gaithersburg, MD, USA). We used the following primer sequences for amplification:

*Cytokeratin14* ( 114 bp ) F: 5ˊ-GTGGCTCTAGCCGCATGTC-3ˊ
  R: 5ˊ-AGCCACTTCCAACCGCA-3ˊ
*Filaggrin* ( 125 bp ) F: 5ˊ-CAGGTGCCAGAACCAACAC-3ˊ
  R: 5ˊ-AAGCAGCATGACCAGTTCC-3ˊ
*Involucrin* ( 200 bp ) F: 5ˊ-CATTCGGAGAAGCAGCCACAG-3 ˊ
  R: 5ˊ-TCCTTCTGGTGCTGTCCCA-3ˊ
*GAPDH* ( 101 bp ) F: 5ˊ-TGCTGGTGCTGAGTATGTCGTG-3ˊ
  R: 5ˊ-CGGAGATGATGACCCTTTTGG-3ˊ

SYBR Premix Ex Taq (Takara Bio Inc., Otsu, Shiga, Japan) was used according to the manufacturer’s protocol. The achieved data was analyzed using threshold cycle (Ct). The formula 2^-ΔΔCt^was used to calculate relative mRNA levels, in which the formula 2^-ΔΔCT^was used as ΔCT=CT of target gene-Ct of housekeeping gene (normalization) and ΔΔCT=ΔCT of co-culture˗ΔCT of control (27). Negative (Day 0 ASCs without any treatment with growth factors or co-culture) and positive controls (keratinocytes) were also used (data not shown). 

### Immunofluorescence analysis

Cells from the epidermal construct were fixed with 4% paraformaldehyde (Sigma, USA) for 20 minutes, rinsed with PBS, and incubated with triton X-100 (Merck, USA) for 10 minutes, rinsed again, then incubated with 3% bovine serum albumin (BSA, Sigma, USA) for 2 hours to block any non-specific binding. The cells were stained with primary antibody against pan-cytokeratin (1:100, Abcam Inc., USA) for 2 hours at 4˚C. Then, the specimens were rinsed three times with PBS and incubated with goat anti-mouse fluorescein isothiocyanate (FITC)-conjugated secondary antibody (1:150, Sigma, USA) for 1 hour. Nuclear staining was performed with 4´,6-diamidino-2-phenylindole (DAPI, 1:400, Sigma, USA) for 15 minutes at room temperature. Finally, the epidermal equivalents were rinsed three times with PBS and mounted upside down on a glass slide, then examined by an invert florescent microscope (IX 71, Olympus, Japan). The corresponding negative controls were set using secondary antibodies without the addition of primary antibodies. Therefore, any observed fluorescence resulted from the nonspecific binding of secondary antibody to the sample (27). 

### Morphological evaluation

We chose SEM for morphological analysis. The epidermal equivalents were rinsed three times with PBS and fixed in 2.5% glutaraldehyde for 2 hours. The specimens were further rinsed in PBS, then dehydrated in increasing concentrations of ethanol (10, 20, .., 90, 95, and 100%) for 10 minutes at each concentration, and finally air-dried overnight prior to analysis. 

### Statistical analysis

All data are presented as mean ± SEM from five independent experiments each performed in triplicate. Statistical analysis was performed using SPSS software (version 21.0, SPSS Inc., USA). One-way ANOVA was used to analyze the mean values statistically at a statistical significance of P<0.05. 

### Results

**Fig.2:**
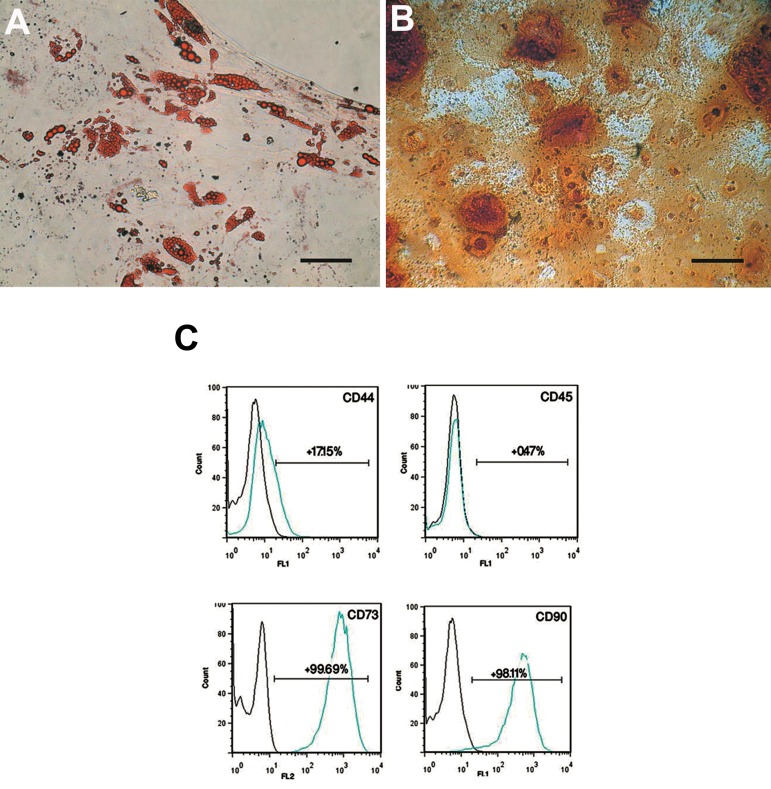
Differentiation potential assay of adipose-derived stem cells (ASCs). A. Adipogenic differentiation shown by oil red O which stained the fat vacuoles inside the cytoplasm, B. Mineralization following osteogenic differentiation as visualized by alizarin red S staining (scale bar: 20 μm), and C. Immunophenotyping of passage 4 ASCs. Histograms indicate the positive mean value of each marker.

After 3 weeks of osteogenic differentiation, mineralization was assessed by alizarin red S staining (Fig.2A). Intracellular lipid vacuoles were visualized as red spots with oil red O staining after two weeks in adipogenic medium (Fig.2B). Flow cytometric analysis of passage 4 ASCs showed expressions of CD73 and CD90 in the majority of cells. Only a few ASCs expressed CD44. CD 45 was expressed in a few cells (Fig.2C). 

**Fig.3:**
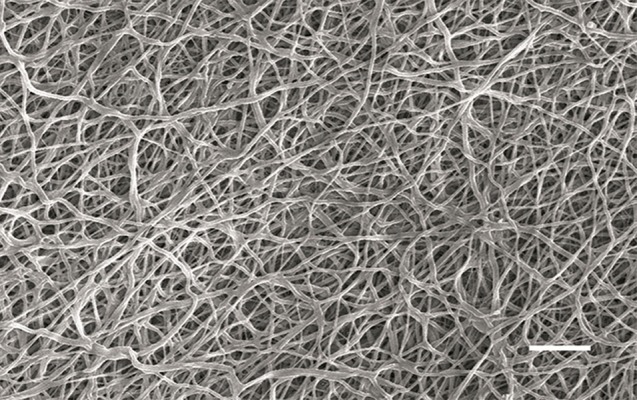
Scanning electron microscope (SEM) image of electrospun polycaprolactone (PCL) matrix that shows randomly-oriented nanofibers (scale bar: 10 μm).

**Fig.4:**
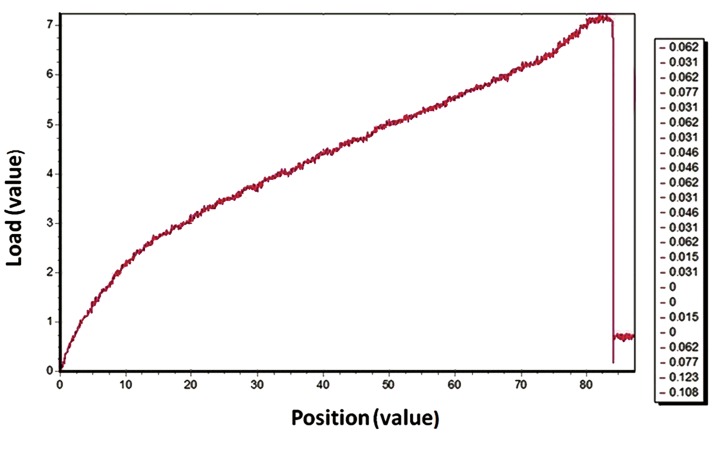
Stress–strain curve of polycaprolactone (PCL) nanofibers under tensile loading.

SEM micrographs of the electrospun PCL matrix showed a porous, beadless randomized nanofibrous structure. Measurement of the fiber diameters has revealed that they were nanosized and mostly under 1500 nm (75%, Fig.3). Figure 4 shows the stress-strain curve of PCL nanofibers under tensile loading. The 3D PCL matrix usually breaks from the application of stress by means of stretching the membrane. Table 1 shows the mechanical properties of the PCL matrix.

**Fig.5:**
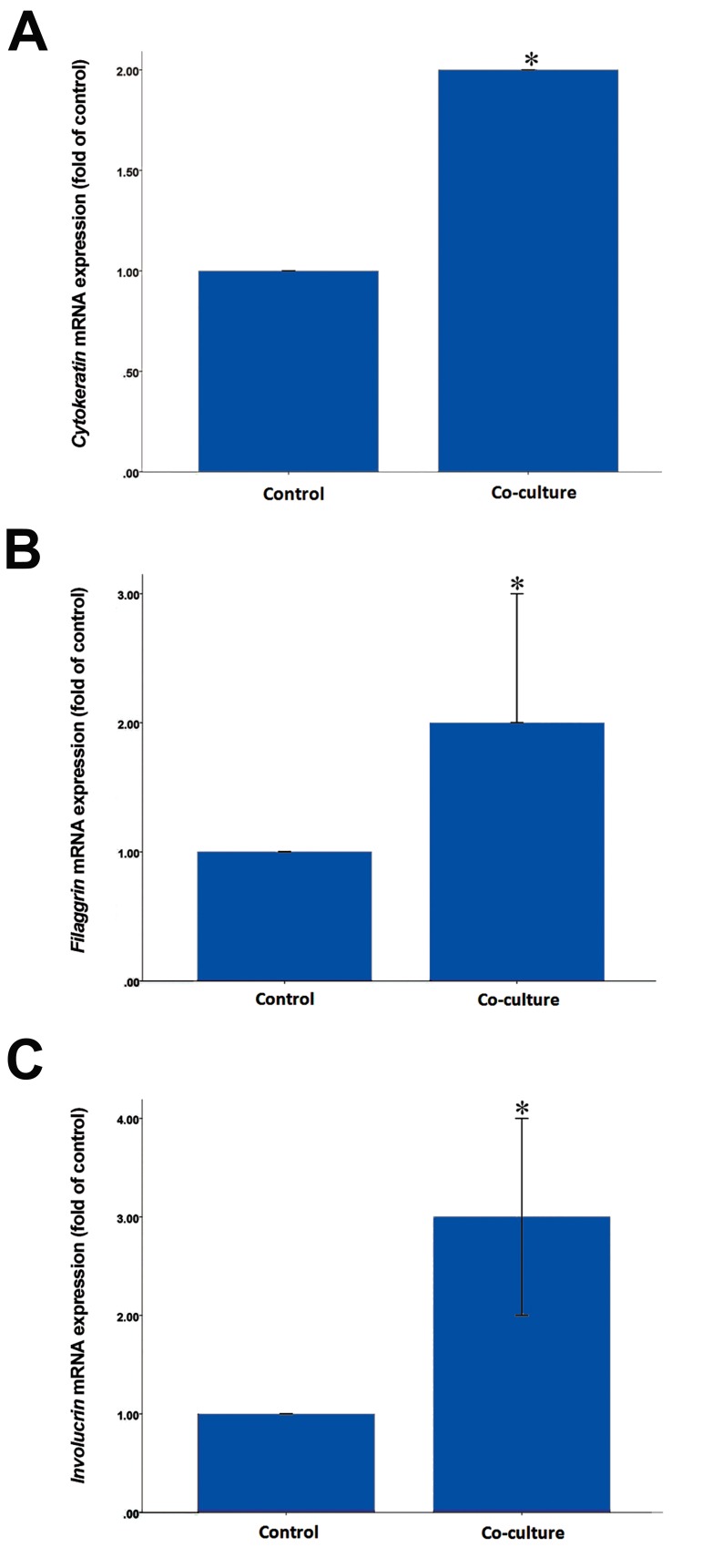
Relative expression of *Cytokeratin14, Filaggrin,* and *Involucrin* genes in the experimental groups. A. Cytokeratin mRNA expression increased 2.1 ± 0.01 fold in the co-culture group compared to the control group, B. *Filaggrin* mRNA expression increased 2.73 ± 0.02 fold in the co-culture group compared to the control group, and C. *Involucrin* mRNA expression increased 3.75 ± 0.03 fold in the co-culture group compared to the control group. Data are the mean ± SEM of 5 separate experiments. *; P<0.001 compared with the control group.

Quantitative real-time RT-PCR demonstrated that mRNA expression of *Cytokeratin14, Filaggrin,* and *Involucrin* increased significantly in the co-culture groups compared to the control groups (P<0.001). These findings indicated that the organotypic co-culture technique upregulated the expression of the aforementioned genes (Fig.5). 

**Fig.6:**
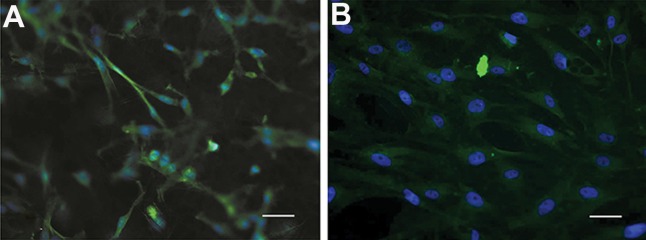
Fluorescence microscope images of A. Control and B. Co-culture groups. Note the cobble-stone appearance of differentiated ke- ratinocyte-like cells co-cultured with dermal fibroblasts (DFs) (scale bar: 20 μm).

ASCs that differentiated on PCL nanofibers in both groups were stained for pan-cytokeratin. Cells on the control scaffolds had either a fibroblastic-like phenotype, typical for ASCs morphology or round morphology that indicted earlier stages of keratinocyte differentiation (Fig.6A). Differentiated cells had a polygonal and cobblestone appearance in the co-culture group (Fig.6B). The remarkable differences in the rate of differentiated cells and their morphology between the co-culture and control groups indicated that DFs had the capability to influence ASCs and induce keratinocyte differentiation in this organotypic co-culture system with PCL matrices. 

**Fig.7:**
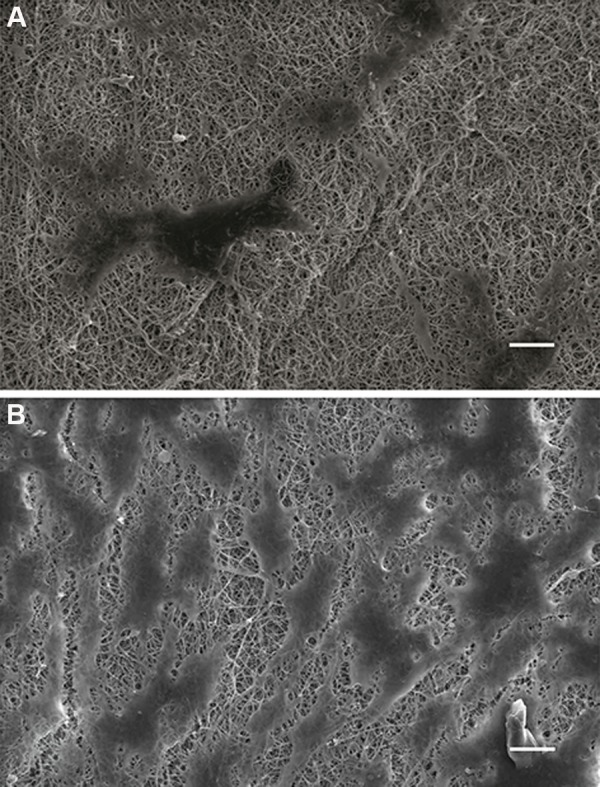
Scanning electron microscope (SEM) image of differentiated keratinocyte-like cells cultured on electrospun polycaprolactone (PCL) matrices. A. Differentiated adipose-derived stem cells (ASCs) appeared to be large with multiform cytoplasm in the control group (scale bar: 50 μm) and B. Differentiated ASCs in the co-culture group displayed cobble-stone shapes that confirmed the role of dermal fibroblasts (DFs) upon further differentiation of ASCs into keratinocyte-like cells (scale bar: 20 μm).

Based on SEM images, differentiated ASCs that interacted with DFs had squamous, cobblestone morphologies, considered to be the specific morphology for keratinocytes under *in vitro* conditions. In the absence of DFs, the differentiated ASCs exhibited a large, flat morphology and were less squamous (Fig.7). 

**Table 1:** Basic characteristics of the electrospun polycaprolactone (PCL) nanofibrous matrix


	Elongation at break (mm)	Load at break (n)	Tensile strength (mpa)	Strain at break	Tensile strain %	Elastic modulus (mpa)

PCL matrix	86.20 ± 1.97	7.82 ± 0.58	4.62 ± 0.34	3.25 ± 0.29	325.29 ± 28.80	1.43 ± 0.23


mm; Millimeter, n; Newton, and mpa; Mega pascal.

## Discussion

The organotypic culture represents a synthetic approach. However, the 3D, high density culture is regenerated from isolated cell lineages that are subsequently recombined, after which their interaction is studied and their response to exogenous stimuli characterized. The exogenous stimuli may be regulatory hormones, nutritional conditions, or xenobiotics. In each case the response is likely to be different from the responses of an isolated pure cell type grown at a low cell density (9, 10, 14). In this study, we have developed an organotypic co-culture skin model that used ASCs and DFs as interactive cells on PCL matrices which created a contributory and inductive microenvironment that favored epidermal differentiation of ASCs. Differentiation of ASCs into non-mesenchymal lineages has always been a significant concern. Numerous studies aimed to create fully differentiated keratinocyte-like cells from ASCs by evaluating various stimuli and conditions that influenced their epidermal differentiation (19-24). The present study showed that DFs could operate as a potent stimulator for ASCs differentiation into keratinocyte-like cells. Our results supported previous reports that verified the key roles of DFs in skin development. It has been shown that reepithelialization and tissue homeostasis of the skin are regulated by epithelial-mesenchymal interactions (30-35) and that keratinocytes induce DFs to produce growth factors which in turn motivate keratinocyte proliferation and keratinization (31-33). Several studies reported that DFs played a specific role in epidermal homeostasis and wound healing by contributing to the regulation of keratinocyte proliferation and differentiation (35-38). Co-culture of keratinocytes with fibroblasts promoted further keratinocyte differentiation (33, 35, 39, 40). In addition, research showed that fibroblasts induced keratinocyte proliferation and differentiation in a 3D organotypic co-culture model by the secretion of periostin (37).

By taking into consideration the requirements of scaffolds for tissue engineering applications, synthetic and natural scaffolds and matrices can be designed and tailored to obtain a wide range of unique combinations with acceptable mechanical, degradation, hydrophilicity, and biocompatibility properties (40, 41). Electrospun nanofibers can mimic the native skin ECM environment and promote epidermal differentiation of stem cells. Therefore, they are promising substrates for advanced skin tissue engineering (42-44). Based on these studies, nanofibers have been chosen as substrates for setting up the organotypic co-culture. Furthermore, PCL, because of its biocompatibility and biodegradability is extensively investigated for tissue engineering applications. PCL is non-toxic in nature and cytocompatible with several body tissues, which makes it an ideal material for tissue engineering (45). PCL contains flexible mechanical properties (Young’s modulus, elasticity, tensile strength, and elongation at break value) as has been confirmed by our study. PCL fibers are used as the material of choice for surgical sutures and wound dressing (45-47). Thus, PCL can be a good candidate for long-term tissue engineering implants.

Of note, electrospinning technology can act as an effective tool for the successful preparation of PCL scaffolds with different topographical structures. The morphology and architecture of an electrospun structure can be designed similar to that of the natural ECM (6, 28, 45, 46). The main feature of nanofibers is the high surface area to volume ratio which can support cell attachment, proliferation, migration, and differentiation (44). Superior mechanical properties and high porosity make the nanofibrous scaffolds and matrices an ideal substrate for tissue engineering applications (48-52). For this reason, we have used PCL nanofibers in the present study to establish a novel skin co- culture model. Until now, culturing stem cells or keratinocytes in an optimized induction medium on a contractible fibroblast-embedded collagen gel with an air-liquid interface has been considered the common technique for an organotypic co-culture (9, 10). For the first time, we cultured ASCs and DFs on nanofibers in order to create dermal and epidermal equivalents which were combined in a manner similar to Apligraf® (a commercial bilayered construct that uses DFs and keratinocytes). The present study was inspired by the work of Morris et al. (53) who fabricated a two-sided biphasic nanofibrous scaffold. They co-cultured epithelial and fibroblast cells onto the apical nanofiber phase and the basal microfiber phase of a biphasic scaffold, respectively, to provide an optimized 3D platform that mimicked the native microenvironment for cell interactions in an airway structure.

DFs play a key role in the deregulation of keratinocyte growth and re-epithelialization. They mediate keratinocyte proliferation and differentiation via JUN-dependent expression of genes in fibroblasts through paracrine mechanisms (30-35, 54). In the current study, the architecture of PCL nanofibers has made them suitable for diffusion of these paracrine factors of fibroblast to differentiating ASCs. Previously, the epidermal keratinocytes sheet was superimposed on a collagen gel that contained DFs to construct an organotypic model of mouse skin at least for two weeks (9, 10). However, major drawbacks of organotypic co-culture in this form limited the use of these substitutes and included the time-consuming production and early clinical failures because of delayed vascularization of the wound bed or the xenogenic origin of their components. The present study highlighted two important points: i. PCL was an inexpensive, non-toxic material which could be implanted alone or combined with other materials and polymers without any of the above limitations, ii. The use of stem cells has offered the potential to significantly improve the clinical outcome, especially if used in autologous transplantation regimens. With stem cells, the problems with transplant rejection do not exist and ethical objections are avoided (55). Adipose tissue is a ubiquitous, rich source of multipotent stem cells (ASCs) easily accessible in large quantities by an inexpensive, minimally invasive procedure. They can provide a high amount of stem cells considered essential for tissue engineering applications (19, 20, 55).

A 3D co-culture model with filter well inserts, developed by both ASCs and human DFs, was used to investigate the effect of DFs on expression of keratinocyte markers in ASCs in the optimization of the *in vitro* system at the air-liquid interface (39). The study investigated the effect of both DFs and ECM on ASCs differentiation to keratinocytes. However, this attempt was not possible for use in clinical applications. In contrast, the current co- culture skin model could be reproduced in future studies that use different material substances in order to choose the best substances, both functionally and economically. In addition, its epidermal equivalents can be used for implantation purposes in studies on animals and humans.

## Conclusion

3D organotypic co-culture techniques that contain DFs and ASCs seem to be effective for epidermal differentiation of ASCs, most likely by secretion of diffusible specific growth factors and cytokines through a paracrine mechanism. This technique may be applicable for epidermal differentiation of stem cells in future works. This construct probably has the capability to be transplanted to a wound site for healing in future research on skin regenerative medicine.
